# Pembrolizumab-Induced Hypophysitis in the Setting of Renal Cell Carcinoma

**DOI:** 10.7759/cureus.82940

**Published:** 2025-04-24

**Authors:** Kassandra Ogbodu, Mukul Sharda, Naisarg B Vanani, Pinky Jha

**Affiliations:** 1 Internal Medicine, Medical College of Wisconsin, Milwaukee, USA

**Keywords:** adrenal insufficiency, endocrinology, hypophysitis, immune checkpoint inhibitors, pembrolizumab

## Abstract

Immune checkpoint inhibitors (ICIs) like pembrolizumab have revolutionized oncology but are associated with immune-related adverse events, including rare cases of hypophysitis. Pembrolizumab-induced hypophysitis typically presents as an isolated adrenocorticotropic hormone (ACTH) deficiency. However, atypical cases with panhypopituitarism and pituitary enlargement have been reported. Here, we describe a unique case of delayed-onset pembrolizumab-induced hypophysitis in a patient with renal cell carcinoma, highlighting the need for continued vigilance in recognizing endocrine complications even months after therapy cessation.

A 55-year-old female with renal cell carcinoma, treated with left nephrectomy and pembrolizumab, presented with weakness, orthostatic hypotension, nausea, and vomiting. She had undergone multiple prior evaluations without a clear diagnosis. On admission, hypoglycemia and hypotension raised suspicion for adrenal insufficiency. Initial endocrine testing revealed low AM cortisol and ACTH levels, suggesting central adrenal insufficiency. A cosyntropin stimulation test confirmed the diagnosis. MRI of the sella showed no pituitary mass but was positive for hypophysitis, attributed to her prior pembrolizumab therapy. The patient was treated with hydrocortisone, resulting in symptom relief.

This case highlights a rare instance of hypophysitis secondary to pembrolizumab, a programmed cell death protein 1 (PD-1) inhibitor, in a patient with renal cell carcinoma. While thyroid dysfunction is more common with ICIs, hypophysitis remains an uncommon complication, especially with anti-PD-1 agents. Our patient’s late presentation, nearly five months after the conclusion of treatment, suggests lasting effects of pembrolizumab on the hypothalamic-pituitary system.

## Introduction

Immune checkpoint inhibitors (ICIs) have revolutionized cancer therapy by enhancing antitumor immunity, particularly through agents targeting programmed cell death protein 1 (PD-1) such as pembrolizumab. While these therapies have significantly improved outcomes across various malignancies, they are associated with immune-related adverse events (irAEs), including endocrinopathies like hypophysitis. Notably, hypophysitis is more commonly linked to cytotoxic T-lymphocyte-associated protein 4 (CTLA-4) inhibitors, with an incidence of approximately 10%, whereas PD-1 inhibitors like pembrolizumab have a reported incidence of less than 1% [1].

The clinical manifestations of pembrolizumab-induced hypophysitis often involve isolated adrenocorticotropic hormone (ACTH) deficiency, presenting with symptoms such as fatigue, hypotension, and hypoglycemia [2]. However, cases exhibiting panhypopituitarism and pituitary enlargement are exceedingly rare. For instance, a case reported early disease onset after single-agent pembrolizumab initiation, accompanied by panhypopituitarism and increased pituitary mass, contrasting with the more typical presentation of isolated ACTH deficiency [3].

In this report, we present a unique case of pembrolizumab-induced hypophysitis in a patient with renal cell carcinoma, characterized by delayed onset of symptoms manifesting three months post-therapy cessation. This case underscores the necessity for clinicians to maintain a high index of suspicion for hypophysitis in patients presenting with nonspecific symptoms, even long after the completion of pembrolizumab treatment.

This article was previously presented as a meeting poster at the 2024 Annual Society of Hospital Medicine Meeting and the 2024 Annual American College of Physicians meeting on April 14th, 2024, and April 19th, 2024, respectively.

## Case presentation

Our patient is a 55-year-old female with a past medical history (PMHx) significant for renal cell carcinoma status post left-nephrectomy and pembrolizumab immunotherapy, asthma, depression, type II diabetes mellitus, essential hypertension, photopsia, postoperative nausea and vomiting, and sciatica who presents with complaints of generalized weakness and malaise. On her arrival, the patient elaborates that she presented to the emergency department after being sent by her primary care physician after worsening weakness, dizziness, nausea, vomiting, diarrhea, food aversion, headaches, and orthostatic hypotension at home with a brief episode of syncope.

Given that the patient’s presenting symptoms during her admission were consistent with symptoms she had been experiencing for 10 months preceding her admission, it is important to review this history as a context for this case. As such, the patient had been admitted with similar complaints nine times since her left nephrectomy, each time to return to the hospital a short time later with similar presenting concerns. The patient reports that she has had an ongoing recurrence of episodes of hypotension similar to the present case (September 2023) since December 2022. These episodes had progressively become worse since her L-nephrectomy in February 2023, most recently presenting with a syncopal episode. Notably, she had been maintained on pembrolizumab as adjuvant therapy since her nephrectomy and had completed three cycles prior to this admission.

Along with the recurrent episodes of orthostatic hypotension, despite the patient’s previously poorly controlled type II DM with a glycated hemoglobin (A1C) of 12 at diagnosis, the patient had been presenting to the emergency department over the preceding four months with hypoglycemia of unexplained etiology, typically in the range of 40-60 mg/dL. Given the protracted and recurrent nature of her symptoms, the patient had been previously worked up both by various emergency departments and subspecialists without any definitive diagnosis or lasting treatment of her symptoms.

This workup included neurological follow-up for dysautonomia, peripheral neuropathy, and diabetic gastroparesis; gastroenterology for 60 lb weight loss in one year along with possible median arcuate ligament syndrome; pain management for myofascial and neuropathic pain; and cardiology for recurrent orthostatic hypotensive episodes. Prior to the current hospitalization, the patient was most recently seen by her cardiologist, where she had been followed for these orthostatic episodes, which were confirmed by a positive tilt table test.

Two months prior to her current presentation, the patient was trialed on fludrocortisone (mineralocorticoid) replacement therapy by cardiology for a three-week course without improvement in symptoms and subsequent hospitalization due to an orthostatic hypotensive episode with syncope. During this last hospitalization, she was discontinued on the fludrocortisone and was initiated on midodrine by her cardiologist as an attempt to control her hypotensive episodes. When asked about her compliance with midodrine on current admission, the patient reported that she stated that the instructions tell her she "has to take it with food," and since she no longer eats meals, she had only been taking 1/3 of the prescribed doses per day.

On admission, the patient was also found to be hypoglycemic and hypotensive with orthostatic vital signs (Table 1). This constellation of symptoms was initially concerning for an adrenal insufficiency picture. This prompted an initial endocrine workup on hospital day 2, which demonstrated a low AM cortisol of 0.9 ug/dL, with a low ACTH at 5.0 pg/mL. Given these findings, concern for central adrenal insufficiency was increased. On hospital day 3, further endocrine evaluation included a cosyntropin stimulation test, which demonstrated a low baseline cortisol of 5.13 µg/dL, with 30- and 60-minute values of 12.3 µg/dL and 16.5 µg/dL, respectively - findings consistent with secondary adrenal insufficiency.

In the setting of her headaches, among other concerns, this endocrinology workup was followed by an MRI sella, which was negative for pituitary masses such as micro- or macroadenomas.

**Figure 1:**
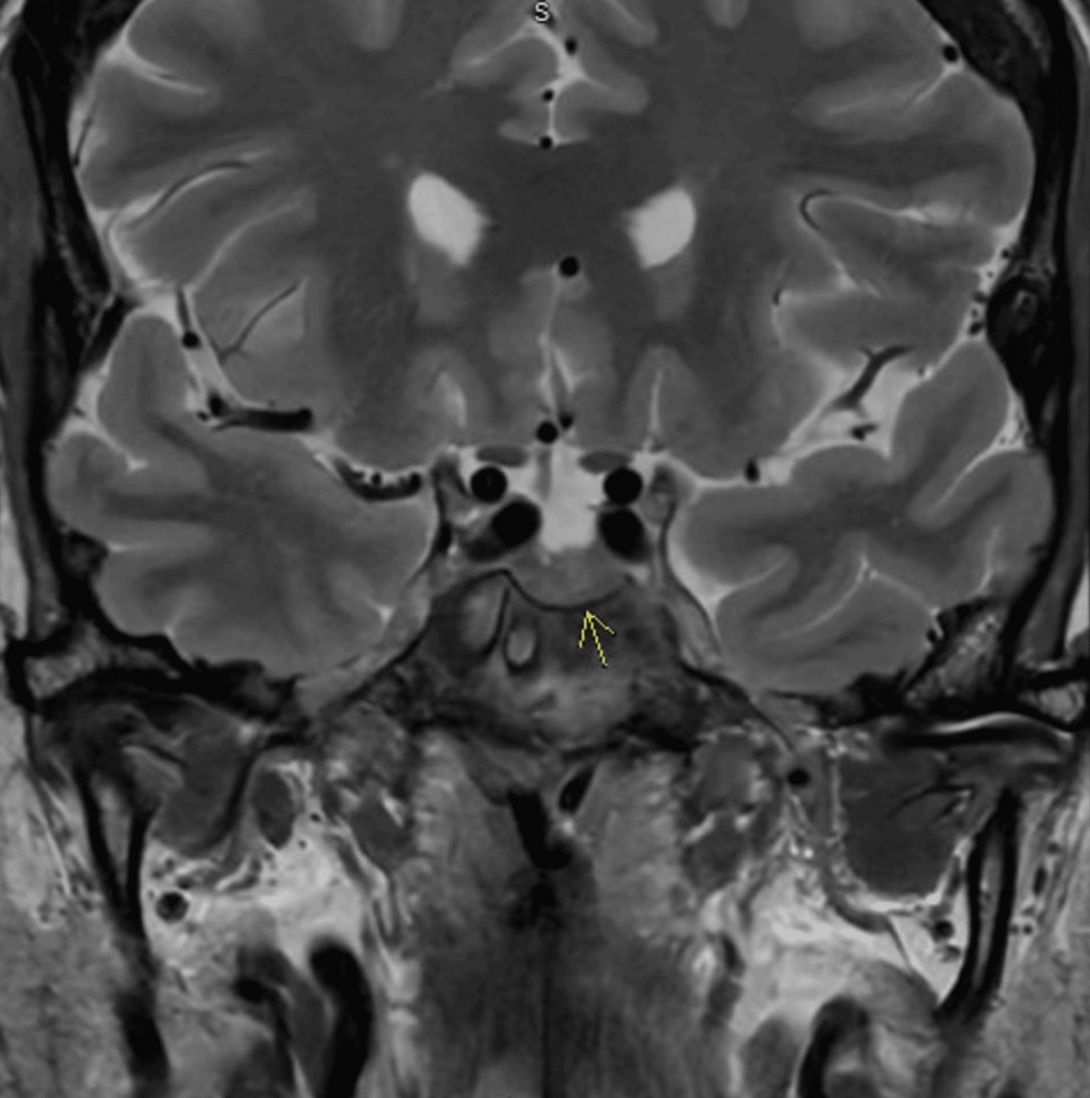
MRI sella with contrast. Arrow points to the area of hypophysitis

The anterior pituitary was normal in size with a slightly concave superior margin and a centrally located 6 mm area of inhomogeneous signal and enhancement on coronal imaging. Given the clinical context, this raised concern for hypophysitis (Figure 1). After discussions with the endocrinology team along with a multidisciplinary literature search, the hypophysitis was ultimately named as a most likely side effect of her prior immunotherapy with pembrolizumab.

The patient was then initiated on adrenal replacement therapy with IV hydrocortisone, on which she was observed inpatient for two subsequent hospital days. After initiation of this cortisol replacement therapy, the patient’s hypotensive episodes resolved with a return to normotensive pressures and negative orthostatic vital signs (Table 1). The patient had begun eating all three meals with a return of her appetite and resolution of her hypoglycemia.

**Table 1:** Pertinent vital signs and labs on presentation versus discharge BUN: blood urea nitrogen

Value	On admission	On discharge	Reference range
Blood pressure (mmHg)	82/45	136/82	< 120 mmHg systolic; < 80 mmHg diastolic
Fasting blood glucose (mg/dL)	63	176	Fasting: 70-99 mg/dL
Sodium (mmol/L)	127	137	135-145 mmol/L
Potassium (mmol/L)	4	4.4	3.5-5.0 mmol/L
BUN (mg/dL)	11	16	7-20 mg/dL
Creatinine (mg/dL)	1.1	0.91	Men: 0.74-1.35 mg/dL; Women: 0.59-1.04 mg/dL

From a subjective standpoint, the patient reported that she felt “20 years younger” and was making “miles” of progress with the physical therapy. The patient was then transitioned to oral hydrocortisone therapy, on which she is to remain for her life given her lack of endogenous hypothalamic-pituitary-adrenal axis. Since her hospitalization, the patient continues to follow up outpatient with the endocrinologist and cardiologist, among her previous subspecialist providers, and has been free of readmission for similar complaints for over one year.

## Discussion

Our patient presents a unique clinical picture of hypophysitis secondary to pembrolizumab, a single-agent PD-1 inhibitor. The observations in this case diverge from the prevailing pattern, as most side effects involving ICIs predominantly affect the thyroid or an isolated ACTH deficiency, with hypophysitis being the least common side effect, even more so in the case of anti-PD1 agents [4]. To the best of our knowledge, this patient represents a unique case, being the first to manifest these symptoms in the context of pembrolizumab monotherapy specifically for renal cell carcinoma, as well as being the first case involving a patient who has undergone both unilateral nephrectomy and adrenalectomy in this context. Interestingly, our patient’s presentation of persistent symptoms three months removed from ending pembrolizumab treatment suggests that the drug can cause lasting effects on the hypothalamic-pituitary system. While PD-1 inhibitors are predominantly administered in conjunction with other targeted therapies and chemotherapy regimens, the patient’s monotherapy with pembrolizumab allowed us to characterize the effects of this medication in isolation [5].

Pembrolizumab, a monoclonal antibody targeting PD-1, enhances T-cell-mediated immune responses by inhibiting the interaction between PD-1 and its ligands (PD-L1 and PD-L2). While this mechanism is effective in reinvigorating antitumor immunity, it can also lead to off-target immune activation, resulting in irAEs affecting endocrine organs such as the pituitary gland [6]. Unlike hypophysitis induced by CTLA-4 inhibitors, which is typically characterized by direct lymphocytic infiltration of the pituitary, PD-1 inhibitor-associated hypophysitis is thought to be mediated by a different immune mechanism. Studies suggest that PD-1 blockade leads to sustained T-cell activation and an increase in autoreactive lymphocytes, which may target pituitary-specific antigens [7]. Additionally, PD-1 inhibition has been associated with increased production of pro-inflammatory cytokines, particularly interferon-gamma (IFN-gamma) and tumor necrosis factor-alpha (TNF-alpha), which can disrupt the function of pituitary cells and contribute to irreversible hormone deficiencies [8].

Furthermore, the pituitary gland exhibits unique immune characteristics, including the expression of PD-L1 on pituitary endocrine cells. The loss of PD-L1-mediated immune tolerance may facilitate targeted autoimmunity against pituitary tissue, ultimately resulting in endocrine dysfunction. Unlike CTLA-4-related hypophysitis, which is often reversible with high-dose glucocorticoids, PD-1 inhibitor-induced hypophysitis tends to cause persistent hormonal deficiencies, as seen in our patient [9].

In our comprehensive review of the literature, we have identified multiple cases and studies illustrating the varied clinical manifestations of pembrolizumab-associated hypophysitis across different contexts. In their 2018 study, Hanna et al. detailed an acute kidney injury precipitated by pembrolizumab-induced adrenalitis and adrenal insufficiency, highlighting the extensive systemic effects of hypophysitis that extend beyond the pituitary gland. This case underscores the broader impact of irAEs on multiple organ systems [10].

Further exploring the severe and diverse complications associated with pembrolizumab, Oristrell et al. (2018) reported a critical incident of cardiac tamponade that occurred in conjunction with adrenal insufficiency, emphasizing the significant cardiopulmonary challenges accompanying endocrine irAEs [11]. Meanwhile, Leiter et al. (2020) uncovered a frequent presence of pituitary autoantibodies in patients suffering from ICI-mediated hypophysitis, suggesting a strong autoimmune component to this adverse effect [12]. Similarly, Oğuz et al. (2021) documented the clinical progression and therapeutic management of an isolated ACTH deficiency, indicating a targeted immune-mediated damage to pituitary cells [13].

Adding to these findings, Rossi et al. (2024) reported a remarkable case of triple endocrine dysfunction where a patient developed concurrent thyroiditis, hypophysitis, and adrenalitis after treatment with pembrolizumab, which exemplifies the extensive endocrine disruption that pembrolizumab can induce [14]. Similarly, Fujimiya et al. (2024) described a case of secondary adrenal insufficiency due to ACTH deficiency in a patient treated for non-small-cell lung carcinoma, further highlighting the specific vulnerabilities within the endocrine system caused by pembrolizumab [15].

The complexity of managing pembrolizumab-related hypophysitis is compounded by its varied symptomatic presentations, often necessitating a multidisciplinary approach for accurate diagnosis and effective treatment. Doodnauth et al. (2021) and Montero Pérez et al. (2022) each described instances where hypophysitis manifested as isolated ACTH deficiency, with symptoms that could be easily misinterpreted as more commonplace conditions. These instances highlight the critical need for hypophysitis to be considered in differential diagnoses, particularly for patients undergoing pembrolizumab treatment and presenting with nonspecific symptoms such as fatigue, hypotension, or hyponatremia [16,17].

The literature also elucidates the long-term impacts of pembrolizumab on the hypothalamic-pituitary axis. For example, Khan et al. (2023) discussed a triad of pembrolizumab-induced irAEs, including hypophysitis, demonstrating that the effects of this treatment could be both varied and simultaneous, affecting multiple systems at once [18]. Fernando et al. (2024) and Hanawa et al. (2024) further reported on the delayed onset of hypophysitis symptoms post-therapy, which necessitates prolonged monitoring and management of patients even months after the cessation of therapy [19,20].

## Conclusions

We report a rare case of single-agent pembrolizumab-induced hypophysitis in a patient with renal cell carcinoma. While pembrolizumab has significantly advanced cancer therapy, its association with serious irAEs such as hypophysitis demands vigilant monitoring and comprehensive management strategies. The existing body of literature not only underscores the complexity of pembrolizumab-induced hypophysitis but also accentuates the urgent need for ongoing research to understand its underlying mechanisms and to develop management protocols that effectively mitigate these risks.
